# Bronchial Foreign Body Alerting of a Bronchial Tumor: The Need of a Follow-Up Radiography

**DOI:** 10.1155/2016/6714351

**Published:** 2016-11-22

**Authors:** Nahida El-Rifai, Samar Shahine, Hassan Sidani, Ali Sabeh Aion, Antoine Deschildre, Marie-Christine Copin

**Affiliations:** ^1^Department of Pediatrics, Makassed General Hospital, Beirut, Lebanon; ^2^Department of Pathology, Makassed General Hospital, Beirut, Lebanon; ^3^Department of Surgery, Makassed General Hospital, Beirut, Lebanon; ^4^Service de Pneumologie Pédiatrique, Hôpital Jeanne de Flandre, CHRU Lille, Lille, France; ^5^Institut de Pathologie, Centre de Biologie Pathologie, CHRU Lille, Lille, France

## Abstract

Lung tumors are extremely rare in the pediatric population, comprising only 0.2% of all malignancies in children. Among them, mucoepidermoid carcinoma (MEC) is even rarer with a reported frequency of 0.1% to 0.2%. MEC is defined by the World Health Organization as a tumor characterized by a combination of mucus-secreting, squamous, and intermediate cell types. We describe the case of a 4-year-old girl who presented with a history of intermittent fever and nonproductive cough of 1-month duration after foreign body aspiration. The chest X-ray showed complete collapse of the left lung. After removal of the foreign body, the lung expanded well after. However, the control chest X-ray done after 5 days showed again complete collapse of the left lung. The biopsy specimen taken during bronchoscopy confirmed the diagnosis of low-grade MEC. Fluorescence in situ hybridization (FISH) confirmed the presence of MAML2 rearrangement. Complete surgical resection with preservation of lung parenchyma was performed. No adjuvant therapy was needed. Repeat bronchoscopy was performed 2 months after surgery and showed no recurrence of the tumor. In conclusion, a remote chest X-ray after removal of a foreign body is necessary to avoid missing a rare serious underlying disease such as MEC. According to the size and the location of the tumor, complete surgical removal is sufficient without additional treatment in case of low-grade tumor. The presence of MAML2 rearrangement confers a favorable outcome and may have long-term implications for the clinical management.

## 1. Introduction

Lung tumors are extremely rare in the pediatric population, comprising only 0.2% of all malignancies in children [1]. Among them, mucoepidermoid carcinoma (MEC) is even rarer with a reported frequency of 0.1% to 0.2%. MEC is defined by the World Health Organization as a tumor characterized by a combination of mucus-secreting, squamous, and intermediate cell types. It usually arises in the parotid and submandibular salivary glands and in the minor salivary glands of the oral cavity and perimaxillary region [2].

We describe the case of a 4-year-old girl with low-grade mucoepidermoid carcinoma of the left main stem bronchus initially treated for bronchial foreign body. The diagnosis was made on bronchial biopsy taken during bronchoscopy and confirmed by the presence of MAML2 rearrangement.

## 2. Case Presentation

**Figure 1:**
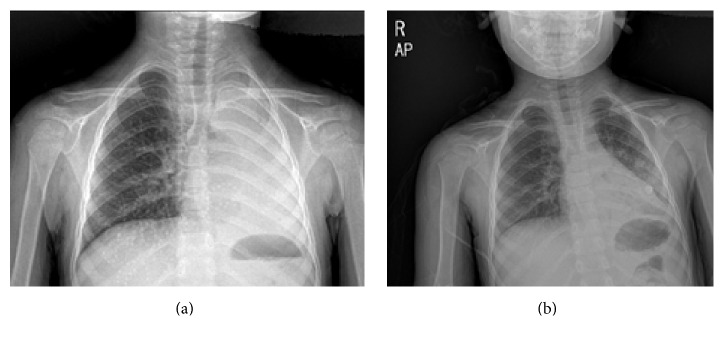
(a) Chest radiograph at admission showing complete collapse of the left lung with mediastinal shift to the left. (b) Chest radiograph showing almost complete expansion of the left lung after bronchoscopy and removal of the foreign body.

A 4-year-old girl was admitted to our hospital for history of intermittent fever and nonproductive cough of 1-month duration. The mother reported a history of choking with seeds 3 weeks before the onset of symptoms. She had no other associated symptoms. The family history was relevant for leukemia from the paternal side. On physical examination, the patient was active, not in distress with diminished breath sounds along the left lung. The rest of physical examination was unremarkable. A chest radiograph showed complete collapse of the left lung (Figure 1(a)). Laboratory investigations showed a white blood cell count of 23000/mm^3^ with 73% of polymorphonuclear cells and 17% lymphocytes, hemoglobin 10 g/dL, hematocrit 30.5%, and platelets 543,000/mm^3^. CRP was 8.5 mg/dL (negative < 0.3). The purified protein derivative (PPD) test was negative.

Rigid bronchoscopy revealed a small part of seed surrounded by a grayish-white polypoid mass adherent to the bronchial wall and completely obstructing the orifice of the left main stem bronchus. Removal of the foreign body and excision of the surrounded polypoid mass were performed. The chest X-ray done after bronchoscopy showed almost complete expansion of the left lung (Figure 1(b)). However, five days later, the patient started to develop high grade fever with persistence of cough. Repeat chest radiograph showed again complete collapse of the left lung. CT scan of the chest with IV contrast revealed complete obliteration of the mid left main stem bronchus by an ill-defined hypodense/enhancing lesion, causing obstructive changes in the left lung with near complete collapse and mediastinal shift to the left with air bronchogram at the base. There were small scattered lymph nodes, subcarinal and left superior mediastinal (13 × 18 mm). The right lung was clear with no pleural or pericardial effusions.

The biopsy specimen taken during bronchoscopy revealed a mixture of tubules and solid areas. The tumor cells were large with round normochromic, often clear central nuclei. They vary from bland clear columnar mucinous, goblet cells to cuboidal expressing CKAE1/3, CK7 diffusely, and CEA focally. Anti-Ki67 was expressed in 10% of the nuclei. P63, CK5/6, CD34, and HMB-45 were absent. Consequently, the diagnosis of low-grade mucoepidermoid carcinoma was confirmed. Complete resection of the mass with bronchotomy and end-to-end anastomosis was undertaken through left posterolateral thoracotomy incision. The regional bronchopulmonary lymph nodes were also removed. The lung expanded after the anastomosis. Intraoperative frozen section analysis revealed tumor-free margins. No metastasis to lymph nodes was observed. Fluorescence in situ hybridization (FISH) confirmed the presence of MAML2 rearrangement. The postoperative course was uneventful. The patient was discharged home 8 days after surgery. Repeat bronchoscopy was performed 2 months after surgery and showed no recurrence of the tumor. Follow-up bronchoscopy after 6 months showed no recurrence.

## 3. Discussion

MEC of the lung is rare in children. It is reported to occur in any age from 3 to 78 years. It equally affects males and females [3]. To the best of our knowledge, this is the first reported case in the pediatric population in Lebanon. Kesrouani et al. reported a 35-year-old pregnant woman with tracheal MEC diagnosed at 27 weeks of gestation [4]. There are no known etiological factors predisposing infants or children to this tumor [5]. However, its occurrence in lungs of patients with congenital abnormalities, including unilateral hypoplastic lung and congenital cystic adenomatoid malformation, has been reported [6–8]. Because of the typical pattern of involvement of large airways, the clinical symptoms and signs include chronic cough, hemoptysis, bronchitis, wheezing, fever, chest pain, and, rarely, clubbing of the fingers [9, 10]. However, the patient may be completely asymptomatic [11]. Our patient presented with history of cough and intermittent fever of one-month duration following a history of foreign body aspiration. The foreign body was removed, but the lung collapse recurred within 5 days. This case illustrates the need to control the chest X-ray after removal of a foreign body to confirm that the foreign body was totally removed, to exclude any persistent complication (atelectasis) and to avoid missing an underlying more serious pathology. Conventional chest radiograph and CT scan generally provide useful information in the evaluation of pulmonary lesions and flexible fiberoptic bronchoscopy constitutes an excellent diagnostic modality. It allows direct visualization of the lesion and biopsies for definitive diagnosis [3]. However, one should be cautious with the biopsies because of the risk of bleeding. In our patient, the chest radiograph showed complete collapse of the left lung. The CT showed an ill-defined hypodense/enhancing lesion occupying the entire left main bronchus with bronchiectatic changes at the base with small scattered lymph nodes.

MEC of the lung may be surgically treated by lobectomy, sleeve resection, local resection, segmental resection, or bronchotomy [9, 12, 13]. This may be done by thoracoscopy or via thoracotomy depending on the location and extent of the lesion [14]. Usually, endoscopic resection is not recommended because of difficulty in controlling hemorrhage and risk of incomplete resection [15]. Recently, Kesrouani et al. reported successful treatment of tracheal MEC by Argon plasma coagulation with no recurrence of the tumor after 5 years [4]. Histological grade, tumor staging, and complete tumor resection are important prognostic indicators [16–18]. Moreover, Zhu et al. demonstrated recently that the presence of MAML2 rearrangement within the tumor was associated with longer overall survival and disease-free survival in pulmonary MEC patients. MAML2 is present in 50% of cases of MEC and it is specific to this tumor. Histologically, this rearrangement is in general recognized to be within cases of low and intermediate grade [19].

In our case, complete resection of the tumor was performed with preservation of the rest of the lung. Since the tumor was of low grade, no adjuvant treatment was administered. The presence of MAML2 rearrangement confers a favorable outcome. Repeat bronchoscopy was done 2 months after the resection of the tumor and showed no recurrence and was scheduled after 6 months.

## 4. Conclusion

In patients with foreign body aspiration, a follow-up chest X-ray is necessary to avoid missing a rare serious underlying disease. In our case, this attitude allows reaching the diagnosis of mucoepidermoid carcinoma (MEC), a rare and exceptional tumor in pediatric age group. Definitive diagnosis of MEC is usually on biopsy specimen taken during bronchoscopy. The presence of MAML2 rearrangement confers a favorable outcome and may have long-term implications for the clinical management. According to the size and the location of the tumor, complete surgical removal is sufficient without additional treatment in case of low-grade tumor.

## Abbreviations

CRP: C-reactive protein
MAML2: Mastermind-Like 2
MEC: Mucoepidermoid carcinoma.
